# Seven pillars for ethics in digital diagnostic assistance among clinicians: Take-homes from a multi-stakeholder and multi-country workshop

**DOI:** 10.7189/jogh.10.010326

**Published:** 2020-06

**Authors:** Lucie Laflamme, Lee Alan Wallis

**Affiliations:** 1 Department of Global Public Health, Karolinska Institutet, Stockholm, Sweden; 2 University of South Africa, Institute for Social and Health Sciences, Johannesburg, South Africa; 3 Division of Emergency Medicine, Faculty of Medicine and Health Sciences, Stellenbosch University, Bellville, South Africa; 4 Division of Emergency Medicine, Faculty of Health Sciences, University of Cape Town, Cape Town, South Africa

Digital technologies can significantly speed up interventions addressing major global health issues, from malnutrition and sanitation to disease and injury. One field of application gaining interest is that of diagnostic assistance to clinicians, where the need in health care delivery globally and the potential for cost-saving are tremendous. Timely, accurate diagnosis is key in the reduction of unnecessary referrals, adequate utilization of resources, and better patient outcomes. Additional benefits of digital assistance are the reduction of professional isolation and better recruitment and retention of staff in remote areas. Medical fields explore these applications in myriad ways [1]. Some modernize a procedure more-or-less in place by tradition (eg, dermatology and radiology), while others revolutionize their practice with the use of extra-laboratory microscopy assistance (eg,“lab-on-a-chip” for pathology or ophthalmology) or artificial intelligence to obtain highly accurate diagnosis (eg, oncology). The health sector has new and powerful means to enhance the readiness of services and to deal with conditions like infections (eg, malaria, tuberculosis, and HIV) or non-communicable diseases [2].

While it promotes the establishment of more equitable health care systems globally, digital health fosters tangible ethical concerns pertaining to human rights like patient autonomy, safety, and justice [3-6]. The concerns most often raised are breaches in patient privacy as individual data are transmitted and circulated, lack of control over secondary use of data, threats to patient safety due to a range of potential errors, and the loss of self-determination among patients and frontline workers when digital routines are put into place.

Recognising the pressing need of tackling those ethical issues, we recently held a multi-stakeholder multi-country three-day workshop with a focus on image-based diagnostic assistance in low resource settings in Sub-Saharan Africa, an obvious target for mHealth applications given their many rural and hard-to-reach communities [7]. We gathered a purposive sample of professionally and geographically spread global mHealth stakeholders (n = 27). The meeting was solution-oriented, consensus-based, and generated lists of actions that address the concerns engendered by mHealth applications by ethical principle (autonomy, justice and safety). Discussions took into account the development, implementation, and scale up phases of an application’s lifecycle. The venue of the meeting inspired the results title of “The Brocher Proposition”.

**Figure:**
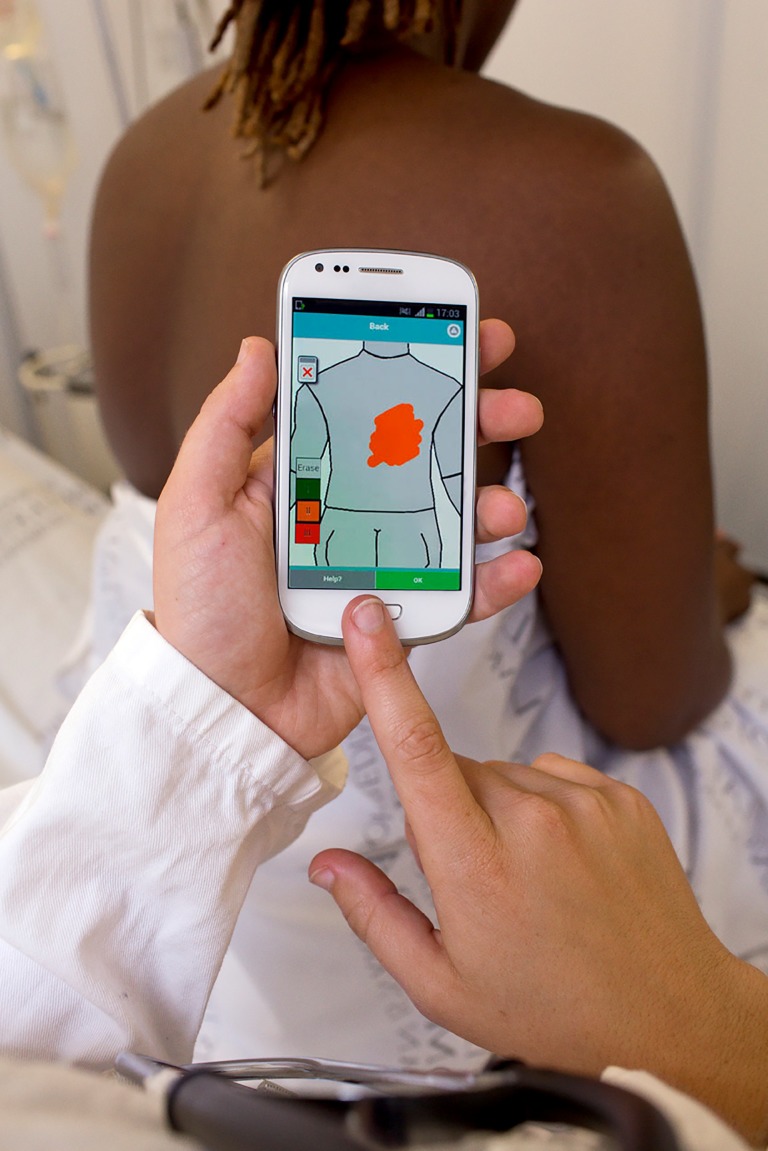
Photo: An app for remote assistance in the diagnosis and management of burn injuries. The authors would like to thank Mattias Lindbäck for the use of his photograph.

**Figure 1:**
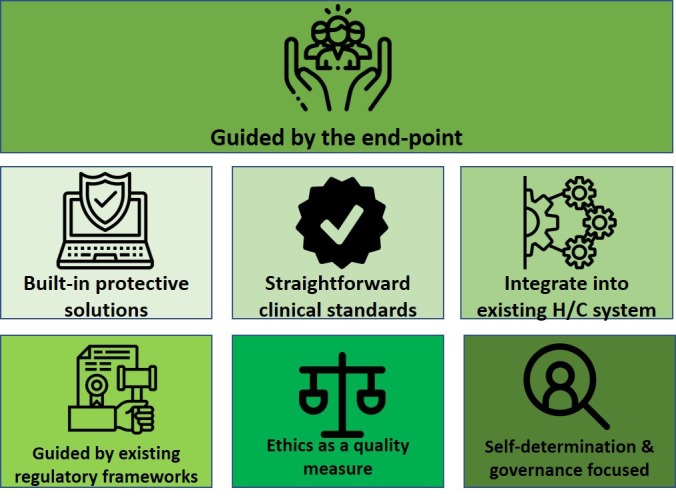
Seven pillars for ethics in digital health.

In this viewpoint, we formulate seven cross-cutting recommendations (presented as pillars in **Figure 1**) derived from that workshop. They underpin the conception of digital health solutions and promote and safeguard good ethical conduct. They find echo in recent publications providing guidance in mitigating negative ethical consequences of digital technologies [5,6,8]. The pillars are listed in **Table 1**, each with a brief explanation.

**Table 1:** Seven pillars for ethics in digital diagnostic assistance among clinicians

Number	Pillar	Clarification
1	Be guided by the endpoint	The ultimate goal of any digital health intervention should be better health. All stakeholders involved, regardless of the competence or perspective they contribute, should bear that in mind.
2	Apply straightforward clinical standards	The gold standard for diagnosis is “bed side” consultation; any compromises on the standard of care delivered must be avoided. Following locally agreed standards that are customised to the health system ensures that clinicians can have confidence in the guidance that is provided.
3	Integrate into existing health care systems	Digital solutions must integrate into current practices in a seamless manner so as to avoid workflow disruption; they must therefore also be relevant in the local health system context.
4	Seek guidance from existing regulatory frameworks	It is essential that already-existing regulations and framework guide the development and implementation process of digital solutions in spite of the need for them to be “locally tailored”.
5	Build-in protective solutions	Stakeholders must be made aware of the potential consequences of errors. Engineers and designers should receive proper guidelines to help build solutions to mitigate the occurrence of errors.
6	Make ethics a quality assurance measure	Routine analysis and follow up mechanisms help foresee and mitigate ethical challenges.
7	Focus on self-determination and governance	Inclusive procedures from development to scale ensure that local stakeholders – including patients themselves – can engage.

***Pillar 1*** is ***“Be guided by the endpoint”***. Not losing track of the ultimate goal is critical because the conception of medical technology and that of digital applications involves many stakeholders — from within and outside the health sector. Digital assistance in health care must remain a means rather than an end, and it should be instrumental to the creation of the best possible conditions to restore, maintain, and promote health. This tenet must be a prerequisite to any decision made, from development all the way to scale-up of digital aids. It should rule not only the development of apps tailored to specific health conditions, but even the use of consumer-oriented apps (eg, WhatsApp) adapted “in house”.

***Pillar 2*** aligns to Pillar 1 and concerns the type of care underpinning digital assistance. It reads ***“Apply straightforward clinical standards”*** and emphasizes that digital health is only an instrument to help facilitate care. The gold standard must remain a face-to-face consultation with the patient. This concern was repeatedly raised at the Brocher meeting, where it was emphasised that digital health solutions should not provide an excuse for substandard care, and that frontline workers must be adequately instructed – and trained accordingly – regarding the acceptable, uncomplicated standards of care in the context of the local health system. Front line clinicians should feel confident about the care that they are advised to provide through the use of digital health; locally agreed standards of care help mitigate this concern.

In the same vein, ***Pillar 3, “Integrate into existing health care systems”***, was forcefully advocated for at the meeting. Any digital solution should be developed and implemented with the local health system at its core, safeguarding seamlessness in the workflow, streamlining productivity, and allowing clinicians to direct energy and effort at patients. Another recurrent consideration was that digital solutions being established using open source platforms, as a means of creating sustainable, agile, and inclusive systems. This pillar will prevent misunderstandings and unnecessary, counterproductive, and disruptive overlaps in the workflow that have implications and consequences for patient privacy and safety.

***Pillar 4*** concerns the need to “***Seek guidance from existing regulatory frameworks”*** rather than formulating new ones to establish an ethical platform to digital health. The primary concern is for the development of apps/devices so they do no harm. Ethical guidelines should be introduced at the time of design of digital technology and app creators should be made aware of the consequences of errors. [6] As of now, as developers are not covered by acts and codes of conduct like the Health Information Portability and Privacy Act (HIPAA) or the International Medical Informatics Association (IMIA) code, and they lack incentive to provide robust security for patient information or to build in “error trackers”. Tying into the international principles behind the frameworks in place pertain to good clinical conduct and has the additional advantage of aiding harmonization within and among health systems.

***Pillar 5*** concerns introducing long-lasting solutions by creating the necessary protective mechanisms from conception rather than sorting problems out on the fly. It is named “***Build in protective solutions”*** and is a promotor of systems that are the least disruptive, that carefully integrate the tasks to be performed and provide the context in which they are used [9], and where stringent security measures prevail. To preserve these systems, ethical guidelines should be introduced at the time of design, and app creators should be made aware of the consequences of errors. To that end, all potential utilization of the system must be explored to ensure the applications cannot be used in an unethical manner. Also, systems must be updated in line with medical progress, as research brings in new management alternatives.

***Pillar 6*** proposes to ***“Make ethics a quality assurance measure”*** as is the case in other fields utilizing digital health. It is essential to plan for monitoring and follow-up, thereby ensuring the responsiveness of a system to the needs of the users and the evolving technological, working, and legislative environments. Analysis and follow-up are means to foresee and avoid ethical concerns. While local regulatory restrictions must be accommodated in the design and usage, by itself this is not enough to ensure ethical use: data must have a responsible guardian and should be protected with appropriate mechanisms; responsibilities for data protection, along with quality improvement mechanisms, must be clearly identified at the outset and updated as needed through the lifecycle of the digital solution.

***Pillar 7*** regards “***Focus on self-determination and governance”.*** The introduction of a third party (machine) into the provider-patient dynamic alters relationships and the balance between them must be restored. Data – whether more or better – cannot take precedence over the patient’s rights. [4]. The Brocher Proposition emphasizes that patient authorization must remain a prerequisite to any digital health intervention, including image-based consultation. It also goes beyond individual self-determination and underlines that all users have a right to engage at all phases of the development of mHealth solutions. Further, it establishes that medical innovations must meet local priorities and rest on locally derived or agreed-upon clinical standards and ethical principles. Governance cuts across all phases of the development of a technological solution and promotes a human rights-based approach to health care [10].

The actual feasibility and, ultimately, even desirability of informed consent was debated extensively during the meeting. The discussions were not straightforward and included questions regarding the levels at which consent must be sought (individual vs community) and whether the community or the individual patient has the final word. The notion of informed consent came across as complex. Tensions may arise regarding whose consent is required (reflecting the conflict between community and individual consent), the complexities of consent for both primary and secondary use of information, and the degree to which patients can be expected to actively engage.

In sum, digital technologies have great potential to contribute to better health and reduced inequalities in health especially in areas and regions of the world where it is needed most [1,2] through enhanced quality and outcome of health care not least as regards remote diagnostic assistance. As a complement to previous significant contributions, the seven pillars derived from the recent multi-stakeholder meeting may guide the set-up of locally relevant systems without compromising basic ethical principles in health care delivery.
